# Spatial Epidemiology of Pediatric Cancer in Romania: A Decade of Persistence, Continuity, and Localized Hotspots (Temporal Trend 2008–2017)

**DOI:** 10.3390/pediatric17060121

**Published:** 2025-11-05

**Authors:** Iulia Daniela Nedelcu, Ion Andronache, Ioannis Liritzis, Helmut Ahammer, Herbert Franz Jelinek, Andreea Karina Gruia, Daniel Peptenatu, Marko Radulovic

**Affiliations:** 1 Interdisciplinary Center for Advanced Studies (CISA-ICUB), Research Institute of University of Bucharest, University of Bucharest, 050663 Bucharest, Romania; iulia.daniela.nedelcu@drd.unibuc.ro (I.D.N.); daniel.peptenatu@geo.unibuc.ro (D.P.); 2 European Academy of Sciences & Arts, 5020 Salzburg, Austria; andronacheion@email.su; 3 Future Technology Institute, South China University of Technology, Guangzhou 510641, China; 4 Gottfried Schatz Research Center Division of Medical Physics and Biophysics, Medical University of Graz, 8036 Graz, Austria; helmut.ahammer@medunigraz.at; 5 Department of Biomedical Engineering, Khalifa University, Abu Dhabi 127788, United Arab Emirates; herbert.jelinek@ku.ac.ae; 6 Faculty of Administration and Business, University of Bucharest, 030018 Bucharest, Romania; karina.gruia@faa.unibuc.ro; 7 Department for Experimental Oncology, Institute for Oncology and Radiology of Serbia, 11000 Belgrade, Serbia; markoradulovic@gmail.com

**Keywords:** pediatric cancer, spatial epidemiology, Geographic Information Systems (GIS), hotspot analysis, temporal trends

## Abstract

**Objective:** Pediatric cancer, though less prevalent than adult malignancies, constitutes a significant public health concern due to its long-term effects on survival, development, and quality of life. This study aimed to investigate spatial patterns and temporal trends of pediatric cancer in Romania over a ten-year period (2008–2017), identifying persistent and emerging geographic hotspots using Geographic Information Systems (GIS)–based modelling and spatial statistics. **Methods:** A national pediatric cancer registry provided by the Ministry of Health was analyzed for cases among individuals aged 0–18 years, categorized by administrative-territorial units (ATUs), ICD-10 codes, sex, and year. Spatial indicators of persistence (recurrent prevalence across multiple years) and continuity (uninterrupted recurrence) were computed. Hotspot analysis was conducted using Local Moran’s I, and trend patterns were assessed through temporal modeling. Additionally, fractal and complexity metrics were applied to characterize the spatial structure and heterogeneity of cancer persistence and continuity across regions. **Results**: Although national pediatric cancer prevalence exhibited a modest decline from 3.57‰ in 2008 to 3.44‰ in 2017, GIS-based spatial modeling revealed stable high-risk clusters in Central and South-Eastern Romania, particularly in historically industrialized counties such as Hunedoara, Prahova, and Galați. These correspond to regions with past heavy industry and chemical pollution. Male children presented a higher frequency of malignant tumors (48,502 cases in males vs. 36,034 in females), while benign and uncertain-behavior neoplasms increased more prominently among females (from 3847 to 4116 cases, compared with 3141 to 3199 in males). Several rural localities showed unexpected prevalence spikes, potentially associated with socioeconomic deprivation, limited health literacy, and reduced access to pediatric oncology services. Regional disparities in diagnostic and reporting capacities were also evident. **Conclusion:** GIS-based spatial epidemiology proved effective in revealing localized, sex-specific, and persistent disparities in pediatric cancer across Romania. The integration of spatial indicators and complexity metrics into national cancer control programs could strengthen early detection, optimize resource allocation, and reduce health inequities. These findings highlight the value of combining geospatial analysis and fractal modeling to guide evidence-based public health strategies for pediatric oncology.

## 1. Introduction

Cancer in children and adolescents is uncommon, yet it remains one of the leading causes of mortality in this age group, second only to accidental injuries [1]. Worldwide, leukemia accounts for roughly 28% of pediatric malignancies, followed by brain tumors (27%), lymphomas (12%) and bone cancers (4%) [2,3,4]. Unlike adult cancers, where lifestyle factors play a major role, childhood cancers are often linked to genetic predisposition and early-life environmental exposures [5,6]. Several neoplasms—such as neuroblastoma, Wilms’ tumor, rhabdomyosarcoma and retinoblastoma—are almost exclusive to childhood, further suggesting distinct etiological pathways [2,3,4].

A critical challenge in pediatric oncology is that the root causes of most childhood cancers remain poorly understood. While genetic factors are important, they alone cannot explain the spatial and temporal variations in prevalence rates. In fact, prevalence was included in this section because it reflects the dataset underlying the study as it was needed to document how prevalence was derived from the recorded cancer cases. There is growing consensus that environmental factors—such as industrial pollutants, agricultural chemicals, air and water contamination, or radiation—may act as triggers or contributors, particularly during vulnerable windows of prenatal and early childhood development [5,6]. However, identifying these factors through traditional epidemiological studies (e.g., case–control) is difficult, expensive, and often relies on imperfect recall of past exposures.

This is where spatial epidemiology, powered by Geographic Information Systems (GIS), becomes a transformative tool. If environmental hazards are influencing cancer risk, their impact should manifest as non-random geographical patterns—specifically, persistent “hotspots” of disease clustered around potential point sources (e.g., factories, mining areas) or diffuse sources (e.g., agricultural regions with pesticide use) [7,8,9,10]. GIS moves beyond simple mapping; it provides a rigorous statistical framework to detect these clusters, quantify their stability over time, and correlate them with geographically referenced data on environmental and socio-economic factors [11,12,13,14,15,16].

Romania presents a compelling case study for this approach. The country has a legacy of heavy industry, intensive agriculture, and regions with documented environmental pollution. Furthermore, disparities in survival rates (approximately 69%, about ten percentage points below Western-European averages [17] hint at underlying inequalities in risk factors, diagnostic access, or care quality [18]. Traditional Romanian cancer studies have focused on aggregated national or regional statistics, which can mask critical local clusters and obscure potential environmental links.

We should mention the methodological advancements and novel contributions of our present study compared to the prior 2023 work [19]. While both studies utilize the concepts of persistence and continuity, there are fundamental differences in methodology, scope, and analytical depth that establish the distinct novelty of our current manuscript. While the [19] study established the value of spatial–temporal analysis for cancer in Romania at a macro level, our present study provides novel evidence by (1) introducing a more reproducible methodology, (2) applying advanced fractal and complexity metrics for the first time in this context, and (3) revealing fine-scale, pediatric-specific risk patterns that were previously invisible. Together, these advancements offer a more precise and powerful tool for public health targeting and etiological research into childhood cancer.

Our study fills a critical methodological and knowledge gap by applying advanced GIS-based spatial–temporal modeling to a decade of Romanian pediatric cancer records (2008–2017) [20,21]. We go beyond simply mapping cases in a single year by introducing two key temporal metrics: persistence (how often an area is a hotspot) and continuity (how long it remains one uninterrupted). This allows us to distinguish stable, endemic high-risk zones—which are strong candidates for having underlying environmental or structural causes—from sporadic, random spikes that might be due to reporting artifacts or chance.

Therefore, the primary objectives of this research are to:

(i) Delineate statistically significant, persistent pediatric cancer hotspots across Romania.
(ii) Quantify the temporal stability (continuity) of these high-risk areas over a ten-year period.
(iii) Lay the essential spatial groundwork for future, targeted investigations by identifying specific localities where the association between disease clusters and potential environmental hazards is strongest.

By exposing these long-term, stable risk zones, our results aim to shift the focus from national-level descriptions to local-level causation. This spatial intelligence is the critical first step in generating actionable hypotheses, guiding future research that can directly measure environmental exposures in identified hotspots, and ultimately informing targeted public health interventions to protect children’s health.

## 2. Materials and Methods

To investigate the spatial and temporal dynamics of pediatric cancer in Romania, we used a decade of registry data and applied a geographic information system (GIS) framework to model persistence and continuity at the national level. The methodological workflow and study area characteristics are detailed below.

### 2.1. Study Area

**Figure 1:**
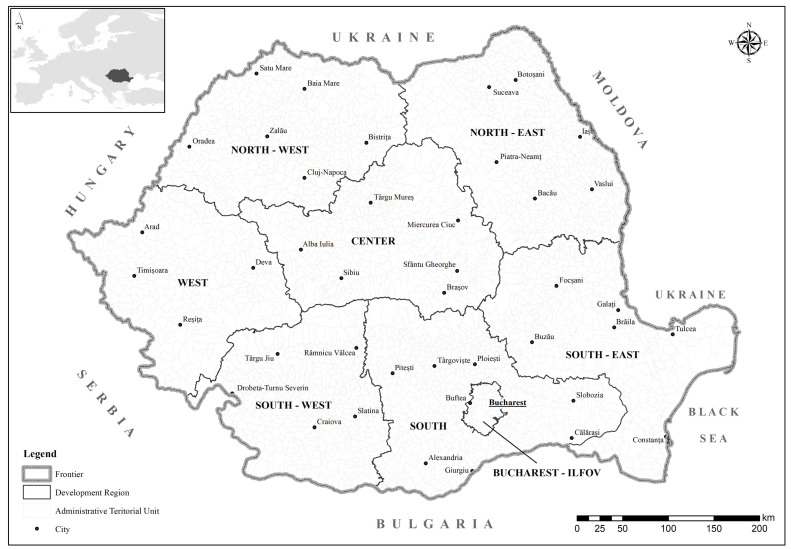
The territorial administrative structure of Romania.

The analysis was conducted at the national level in Romania, a country divided into eight development regions: Northwest, Northeast, Southeast, South, Southwest, West, Center, and Bucharest-Ilfov (Figure 1). Each region includes several counties, totaling 41 counties plus the municipality of Bucharest. Below the county level, the national territory is subdivided into 3181 administrative-territorial units (ATUs), which include municipalities, towns, and communes.

Bucharest-Ilfov stands out by hosting the capital and having the smallest number of local administrative units, in contrast to other regions with more complex territorial structures. This administrative landscape provides a useful framework for examining spatial disparities in pediatric cancer prevalence and persistence.

The study period spans from 2008 to 2017, beginning shortly after Romania’s accession to the European Union. From 2008 onward, Romania adopted standardized cancer-reporting protocols in line with the European Network of Cancer Registries (ENCR), as mandated by Order No. 2027 issued on 26 November 2007 [22]. The Romanian Ministry of Health provided the full dataset used in this study.

### 2.2. Data Sources and Classification

To assess pediatric cancer patterns across Romania, we used a comprehensive database compiled between 2008 and 2017, focusing on individuals aged 0 to 18 years. These data were disaggregated by sex and administrative-territorial unit, allowing spatialized prevalence calculations at local and regional levels.

This is a sufficient length for trends because a 10-year window (2008–2017) is long enough to detect temporal trends and variability while still allowing consistent spatial comparisons.

Also, it has a coding consistency: From 2008 onward, oncology diagnoses and classifications (ICD-10) were applied consistently nationwide, making data comparable across regions and years. This interval is the first full decade captured under the harmonized coding regime.

Last, but not least, inheres data completeness: We capped the series at 2017 to ensure completeness and validation. More recent years may be affected by reporting lags and under-ascertainment, so including them could bias estimates.

The National Cancer Registry of Romania is coordinated by the National Institute of Public Health and collects data reported by hospitals and oncology departments. For pediatric cases, coverage is relatively high due to the concentration of treatment in major university centers (Bucharest, Cluj, Timișoara, Iași), but nationwide completeness is not entirely uniform. Thus, although most diagnosed cases are included in the registry, there may be regional variations determined by the quality and consistency of reporting (data taken by Pediatric oncology centers—Romanian Society of Pediatric Onco-Hematology (SROHP)).

All records were coded using the International Classification of Diseases, 10th Revision (ICD-10), the current standard in the Romanian healthcare system [22].

The dataset includes detailed annual records of both newly diagnosed cases and cancer-related deaths, structured across four analytical levels shown below as Cancer classification based on ICD-10 (Source: WHO, International Classification of Diseases, Revision 10):

Level I—All malignant tumors (C00–C96),
Level II
  - C00–C75: primary malignant neoplasms with specific anatomical locations
  - C76–C80: malignant tumors of ill-defined, secondary, or unspecified sites
  - C81–C96: neoplasms of lymphoid, hematopoietic, and related tissues
Level III—Anatomically distinct categories of primary malignancies:
  - C00–C14: lip, oral cavity, and pharynx
  - C15–C26: digestive organs
  - C30–C39: respiratory and intrathoracic organs
  - C40–C41: bones and articular cartilage
  - C43–C44: melanoma and other skin cancers
  - C45–C49: mesothelial and soft tissue tumors
  - C50: breast
  - C51–C58: female genital organs
  - C60–C63: male genital organs
  - C64–C68: urinary tract
  - C69–C72: eye, brain, and central nervous system
  - C73–C75: thyroid and other endocrine glands
Other Types—Tumors outside classical malignancy:
  - D00–D09: in situ tumors
  - D10–D36: benign neoplasms
  - D37–D48: tumors of uncertain behavior or unpredictable evolution

For the purposes of this study, we considered all pediatric cancer cases in individuals aged 0–18 years, including both sexes. Based on the medical registry data and corresponding population counts, prevalence was calculated using the formula:

*Prevalence* = *A/B* ∗ 1000

where A = All new and existing cases within 0–18 age group during a given time and B = total number of children aged 0–18 during the same period. The total prevalence of females and males (C00-D48) was calculated based on the processed medical database. Estimates were derived solely from the available data; no inclusion or exclusion criteria were applied to individuals registered with cancer.

This methodology ensured comparability across administrative units and facilitated the integration of epidemiological data with spatial models.

The counting rules are as follows:

- If an individual was diagnosed with only one type of cancer each year, they are counted once.
- If an individual was diagnosed with two or more different primary cancers (e.g., leukemia and a brain tumor) within the same year, they are still counted as a single individual in the prevalent count for that year. The unit of analysis is the person, not the diagnosis.

This approach, known as “person-based” prevalence rather than “condition-based” prevalence, is the standard in cancer surveillance for assessing the overall burden of disease in a population. It prevents double-counting individuals with multiple primary tumors, which is a known, though rare, phenomenon in pediatric oncology.

Therefore, the formula above accurately reflects the proportion of the child population affected by cancer at a given time, ensuring the comparability of rates across different administrative units.

### 2.3. Spatial Analysis of Persistence and Continuity

To convert epidemiological data into spatial knowledge with policy relevance, the database described above was analyzed in two key steps: (i) annual classification of administrative-territorial units (ATUs) by cancer prevalence, and (ii) quantification of the temporal stability of high-prevalence zones. The methodological details are as follows.

For each year between 2008 and 2017, pediatric cancer prevalence was sorted in descending order and divided into four quartiles (Q1–Q4), where Q1 represented the 25% of ATUs with the highest values. Based on this classification, two spatial indicators were calculated:

Persistence—the total number of years (0–10) an ATU remained in Q1.

Continuity of Persistence—the longest uninterrupted span (in years) that an ATU remained in Q1.

Both indicators were calculated for total, female, and male cases and classified as shown in Table 1**.**

These indicators offer complementary insights:

- Persistence quantifies how often a territory experiences high cancer prevalence.
- Continuity reveals whether these high values occurred in a cohesive, uninterrupted manner (single interval) or in fragmented sequences (multiple separate periods).

All analyses were performed using Quantum GIS (QGIS) version 3.44, an open-source geographic information system that supports spatial mapping and classification [23]. This spatial modeling enabled:

- Identification of long-term pediatric cancer hotspots;
- Assessment of temporal coherence vs. fluctuation in prevalence.
- Sex-disaggregated comparisons across the national territory.

The findings from this geospatial analysis are presented in Section 3 and further interpreted in the Discussion, with practical relevance for early detection strategies, pediatric cancer surveillance, and equitable health resource allocation.

### 2.4. Statistical Correlation Analysis

To explore potential relationships between the persistence and continuity of pediatric cancer prevalence, we applied the Pearson correlation coefficient to the total, male, and female datasets. This coefficient quantifies the strength and direction of a linear relationship between two continuous variables, with values ranging from −1 (perfect negative correlation) to +1 (perfect positive correlation), and 0 indicating no linear association.

Statistical significance was assessed using a *p*-value threshold of <0.05. This analysis supports the identification of consistent patterns across sex-specific and total cases and complements the spatial models developed in the previous sections.

## 3. Results

Building upon the methodological framework described earlier, this section presents the main findings on the spatial and temporal dynamics of pediatric cancer in Romania between 2008 and 2017. The analysis highlights national and regional trends, sex disparities, tumor typologies, persistence and continuity patterns, statistical correlations, and high-risk localities.

### 3.1. National and Regional Patterns of Pediatric Cancer Prevalence

**Figure 2:**
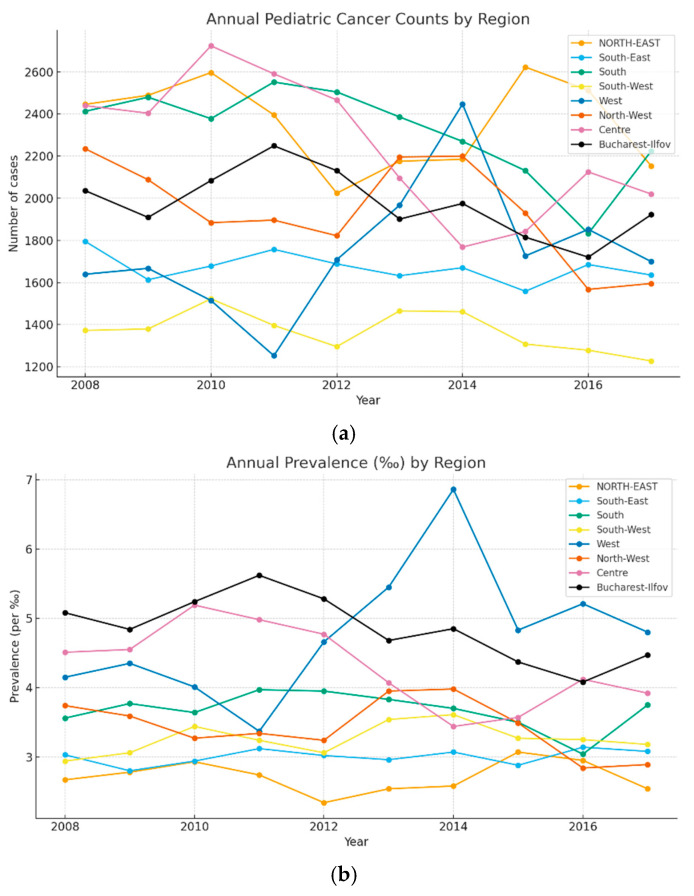

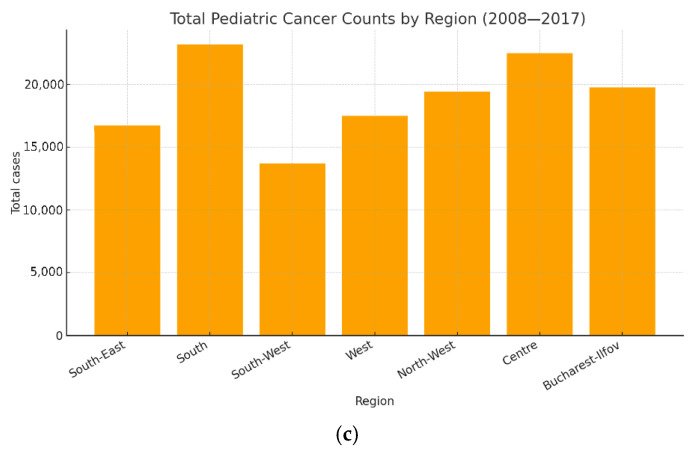
(**a**–**c**) presents the annual evolution of pediatric cancer cases and prevalence rates across Romania’s development regions.

Figure 2a–c presents the annual evolution of pediatric cancer cases and prevalence rates across Romania’s development regions. Over the ten-year study period, the Northeast region reported the highest cumulative number of cases (23,602), while the Southwest region recorded the lowest (13,702). Nationally, the peak prevalence occurred in 2010 (16,381 cases), followed by a gradual decline to 14,475 cases in 2017.

The Bucharest-Ilfov region consistently recorded the highest prevalence, with an average of 4.84‰, although a declining trend was observed—dropping from 5.08‰ in 2008 to 4.47‰ in 2017. Conversely, while most regions showed decreasing prevalence (notably the Northwest, from 3.74‰ to 2.89‰), the Western region exhibited a steady increase, reaching 4.80‰ by 2017. At national level, prevalence peaked in 2014 (3.77‰), then declined to 3.44‰ by the end of the period.

### 3.2. Sex Differences and Cancer Typology

Table 2 summarizes cases by ICD-10 category and sex. Among malignant tumors (C00–C96) boys were more affected (48,502 cases, corresponding to 3.3‰ prevalence) than girls (36,034). Conversely, for other tumors (D00–D48) girls predominated (40,016 vs. 31,837). Our intent here was simply to present the counts of recorded cases, without relating them to the population; there was no specific methodological reason behind this choice.

In fact, Table 3 illustrates the ten-year progression of pediatric cancer cases, by the levels of the International Classification of Diseases. In the realm of malignant tumors including all three primary categories, the male demographic shows a higher propensity for developing one or more cancer types, accounting for 48,502 cases, in contrast to 36,034 cases in females (C00-C96). Notably, both sexes exhibit a reduction in case numbers from the onset of the analyzed period to 2017. In both categories, there was a rising trend in the number of newly reported cases. The initial count in 2008 stood at 6,988 cases, gradually ascending to 7315 cases by 2017.

Both sexes experienced declining malignant tumors across the decade. However, benign and uncertain-behavior tumors (D00–D48) rose—from 6988 cases in 2008 to 7315 in 2017—with a sharper increase among girls.

### 3.3. Most Frequent Tumour Types

**Figure 3:**
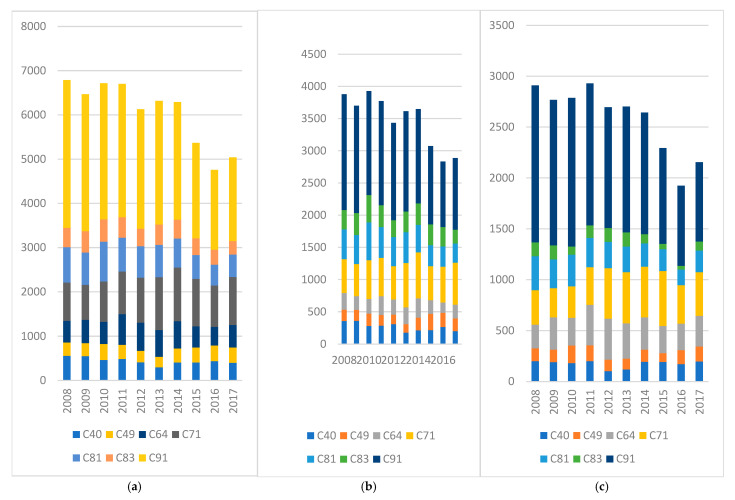
(**a**–**c**) Most frequent malignant tumors by (**a**) total, (**b**) female and (**c**) male cases. C40- Malignant neoplasm of bones and articular cartilage of limbs; C49- Malignant neoplasm of other connective and soft tissue; C64- Malignant neoplasm of kidney, except renal pelvis; C71- Malignant neoplasm of brain; CC81- Hodgkin lymphoma; C83- Diffuse non-Hodgkin lymphoma; C91- Lymphoid leukemia. X axis the years and below the type of malignant tumor and Y axis the number of cases.

**Figure 4:**
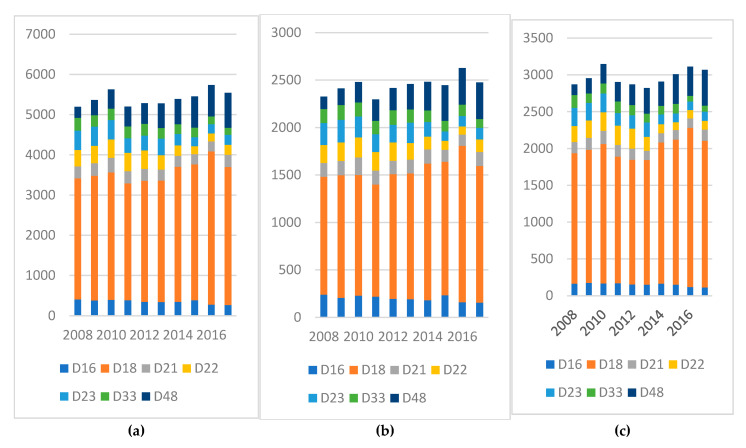
(**a**–**c**) Most frequent benign/uncertain tumors by (**a**) total, (**b**) female and (**c**) male cases. D16—Benign neoplasm of bone and articular cartilage; D17—Benign lipomatous neoplasm; D18—Hemangioma and lymphangioma; D21—Other benign neoplasms of connective and soft tissues; D22—Melanocytic nevus; D23—Other benign neoplasms of the skin; D33—Benign neoplasm of the brain and other parts of the central nervous system; D48—Benign neoplasm of uncertain behavior of other and unspecified sites. X axis the years and below the type of malignant tumor and Y axis the number of cases.

To gain deeper insights into the distribution of childhood cancer, Figure 3 and Figure 4 provide an overview of the most prevalent malignant and benign tumor types within the 0 to 18-year-old population over a decade (2008–2017). Examining Figure 2a, it becomes evident that the highest prevalence is associated with the malignant tumor type C91—Lymphoid leukemia, peaking in 2008 (3343 cases) and subsequently displaying a descending trend. In 2014, it reached 2663 cases, with the lowest recorded cases in 2016 (1805 cases), followed by a slight rise to 1893 cases in the final year of analysis. The next most frequent group of malignant tumors was C71—Malignant Neoplasm of the Brain, which achieved its zenith in 2014 with 1208 cases. Notably, this category exhibited a substantial increase from 795 cases in 2008 to 1078 cases in 2017. C81—Hodgkin’s lymphoma is also notable in the young population, with a peak in 2010 (899 cases). Analyzing cancer cases within the female population (Figure 2b), C91—Lymphoid leukemia takes precedence, with 1543 cases in 2008, 1238 cases in 2013, and 781 cases in 2017. Subsequent prominent tumor categories for females include C71—Malignant neoplasm of the brain, reaching its peak in 2015 with 538 cases, and C64—Malignant neoplasm of the kidney, excluding renal pelvis. Within the male population (Figure 2c), a similar pattern emerges. C91—Lymphoid leukemia stands out, with peak values in 2008 (1800 cases) that decrease to 1112 cases in the final year under analysis. C71—Malignant neoplasm of the brain recorded 712 cases in 2014 and 650 cases in 2017.

Figure 3 and Figure 4 illustrate the most common tumor types identified among pediatric cases in Romania during the 2008–2017 period, distinguishing between malignant neoplasms (C00–C96) and benign or uncertain-behavior tumors (D00–D48).

Among malignant tumors, the most frequently diagnosed was C91—Lymphoid Leukemia, which, despite being the leading type throughout the period, showed a notable decline in annual cases—from 3343 in 2008 to 1893 in 2017. A contrasting trend was observed for C71—Malignant Neoplasm of the Brain, which exhibited an increase over time, peaking at 1208 cases in 2014. Additionally, C64—Malignant Neoplasm of the Kidney was among the most prevalent diagnoses, particularly in the female pediatric population.

In the category of benign and uncertain-behavior tumors, D18—Hemangioma and Lymphangioma emerged as the most frequent. These tumors showed a steady upward trend, increasing from 3014 cases in 2008 to 3436 in 2017. The prevalence was consistently higher among girls, highlighting a sex disparity in this tumor type.

These findings underscore both temporal and sex-specific variations in tumor typology, which may reflect differences in diagnostic practices, biological susceptibility, or environmental exposures.

### 3.4. Persistence and Continuity of Persistence of Pediatric Cancer in Romania

**Figure 5:**
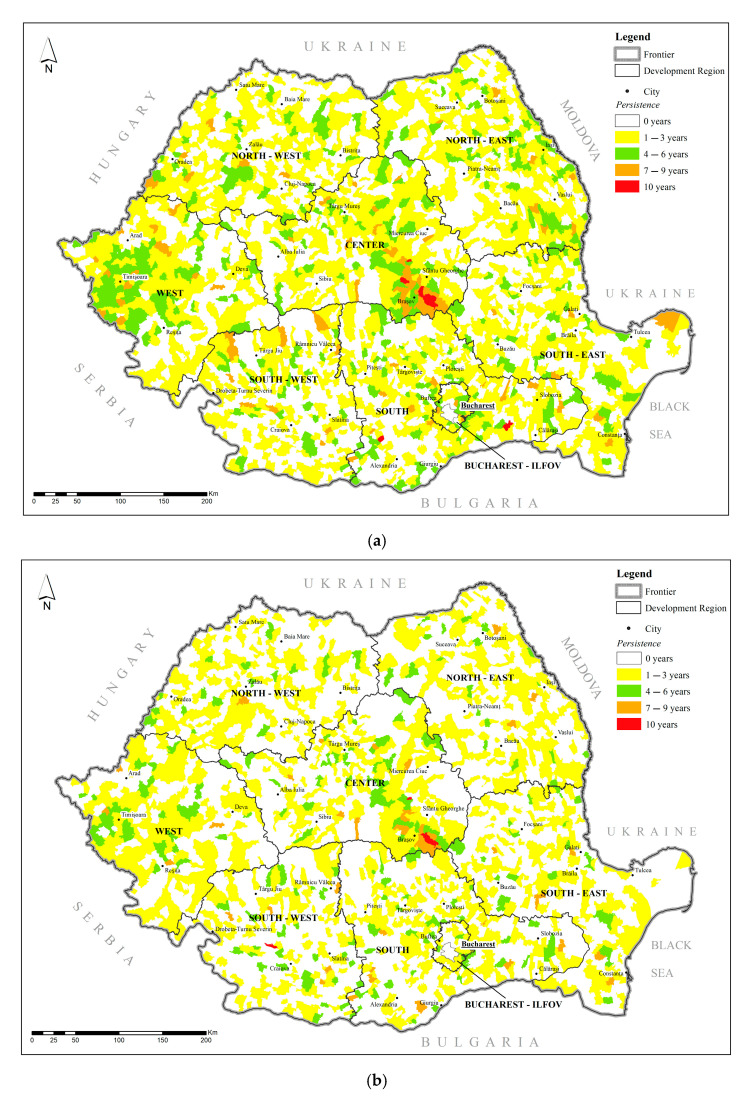

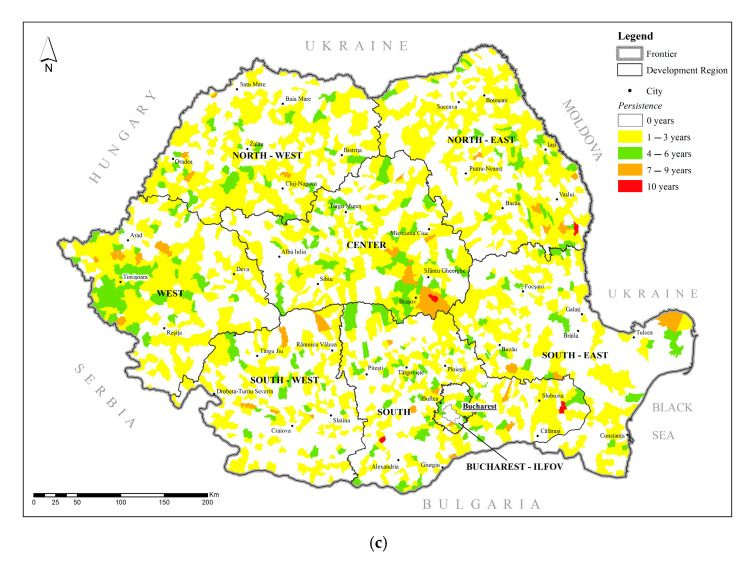
(**a**) Geographical distribution of Persistence—Total. (**b**) Geographical distribution of Persistence—Male. (**c**) Geographical distribution of Persistence—Female. Source: *Ministry of Health*.

**Figure 6:**
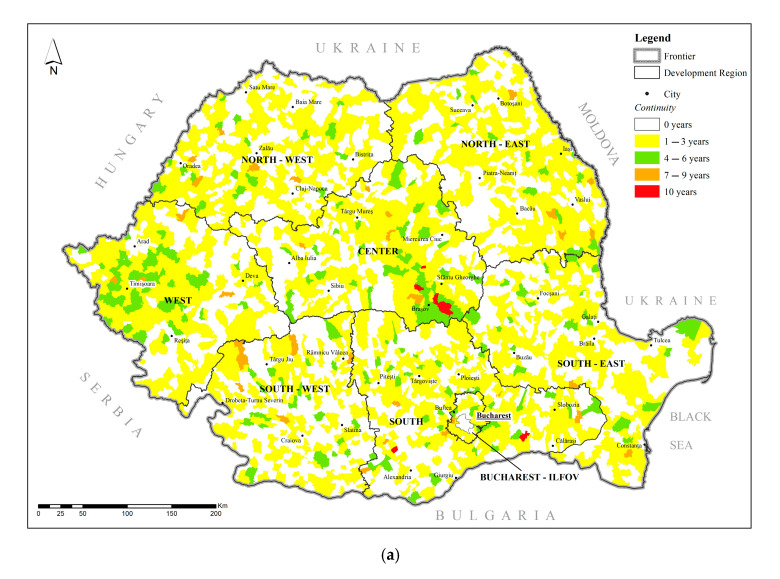

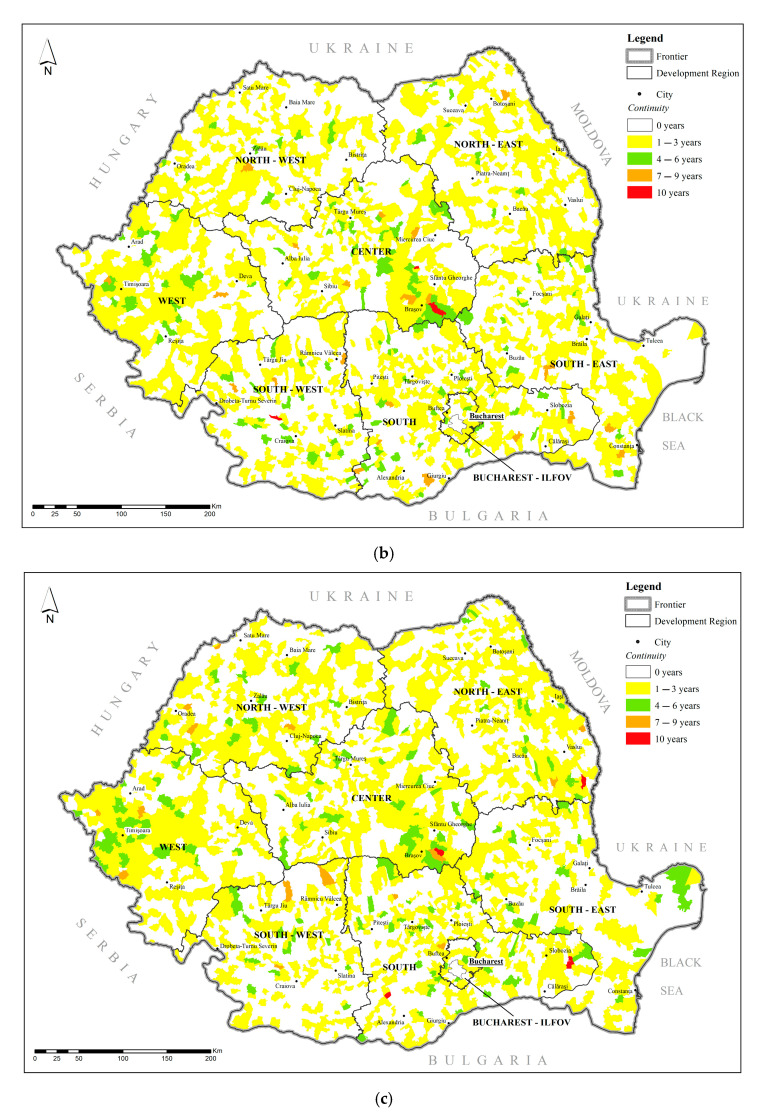
(**a**) Geographical distribution of Continuity—Total. (**b**) Geographical distribution of Continuity—Male. (**c**) Geographical distribution of Continuity—Female. Source: *Ministry of Health*.

Based on the indicators defined in Section 2.3, Figure 5 and Figure 6 illustrate the spatial distribution of pediatric cancer persistence and continuity across Romania’s 3181 administrative-territorial units (ATUs). Persistence refers to the total number of years an ATU remained within the highest quartile (Q1) of cancer prevalence during the 2008–2017 period, reflecting the frequency of elevated values. In contrast, continuity captures the longest uninterrupted span that an ATU stayed in Q1, offering insight into the stability and recurrence of high prevalence over time. Together, these two indicators provide a complementary view of the temporal and spatial dynamics of pediatric cancer burden at the local level.

The classification of these indicators is summarized in Table 3.

For the total population, approximately 44% of ATUs (1391 out of 3181) experienced only sporadic inclusion in Q1 (1–3 years), whereas 139 units exhibited high or maximum persistence (7–10 years) (Figure 4a). These long-term hotspots were predominantly located in Central Transylvania and the Southeast, especially the Bărăgan Plain. Continuity patterns closely mirrored persistence: seven ATUs—distributed across Brașov, Prahova, Galați, and Constanța counties—remained consistently in Q1 throughout the entire decade, while others fluctuated between high and low prevalence, possibly reflecting local changes in exposure or reporting capacity (Figure 5a).

In the male population, persistence maps (Figure 4b) reproduced the total-population pattern, but with a slightly lower number of ATUs in the high and maximum classes (41 vs. 82). The Central region (notably Brașov, Sibiu, Alba) retained the densest cluster of persistent ATUs, followed by isolated zones in Oltenia and Moldova. Regarding continuity (Figure 5b), only three ATUs—two in Brașov and one in Dolj—showed uninterrupted 10-year continuity, while the remainder showed fragmented presence in Q1, suggesting intermittent exposures or inconsistent reporting that may affect boys disproportionately.

In the female population, slightly more ATUs (47) reached high or maximum persistence levels (Figure 4c), compared to males. Beyond Central Transylvania, a pronounced belt of persistent ATUs extended through Southern Muntenia, coinciding with areas of high D18 tumor prevalence (hemangioma/lymphangioma). For continuity (Figure 5c, four ATUs—located in Buzău, Brașov, Suceava, and Teleorman—remained in Q1 across all ten years. The broader pattern shows more scattered moderate-continuity areas, which aligns with the diffuse distribution of benign and uncertain-behavior tumors in girls.

Taken together, Figure 5a–c and Figure 6a–c reveal that long-standing pediatric cancer hotspots are concentrated in counties with a mix of industrial legacies (e.g., Brașov, Hunedoara), high-intensity agriculture (e.g., the Bărăgan Plain), or limited access to specialized care. Persistence maps highlight areas where high prevalence recurs, while continuity maps add nuance by distinguishing stable endemic risk zones from episodic peaks.

### 3.5. Fractal and Complexity Characterization of Spatial Persistence and Continuity

To further characterize the spatial structure and internal complexity of pediatric cancer distribution across Romania, we applied three complementary methods rooted in fractal geometry and complexity theory: the two-dimensional Higuchi fractal dimension (Higuchi 2D) [24,25], Kolmogorov complexity [26,27], and generalized Rényi entropy [28] across a range of orders (q = −5 to +5). These measures were computed on six grayscale spatial maps: persistence and continuity of pediatric cancer prevalence, disaggregated by sex (female, male) and total population.

All six maps were grayscale-normalized prior to analysis to reduce color-based noise and ensure consistent spatial patterning across modalities. Although the visual maps included in this article are rendered in color for clarity, the fractal analysis was strictly applied to the underlying grayscale matrices.

Higuchi 2D provides a scale-invariant descriptor of geometric complexity, with higher values indicating rougher or more irregular surfaces [24,25]. Kolmogorov complexity estimates the compressibility of the spatial configuration—lower values reflect more ordered or repetitive patterns [26,27]. Rényi entropy, computed across multiple q-orders, captures both global and local heterogeneity, with sensitivity to rare events (negative q) or dominant clusters (positive q) [28].

The complete results are presented in Table 4. Notably, maps associated with male prevalence—both for persistence and continuity—show consistently higher Higuchi values (≈2.363) and lower Kolmogorov complexity (≈1.724), suggesting greater structural fragmentation but higher internal regularity. In contrast, total maps show the highest Kolmogorov values (≈2.272), indicating greater overall unpredictability. Female maps generally fall in between, suggesting a smoother but still complex internal patterning.

**Figure 7:**
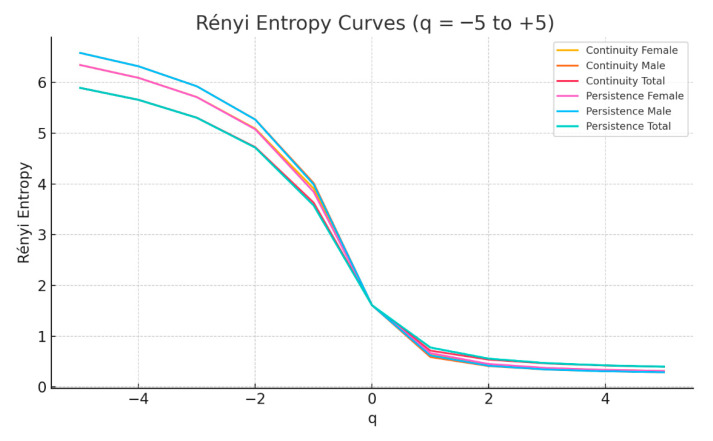
Rényi entropy curves for q = −5 to +5 for the six spatial maps (persistence and continuity by gender and total).

To visualize the multiscale entropy variation, Figure 7 presents the Rényi entropy curves for all six maps across q-values ranging from −5 to +5. These curves decrease monotonically, as expected, and illustrate the entropy signature of each spatial pattern.

In summary, this multiscale fractal analysis provides novel evidence that pediatric cancer hotspots in Romania are not only persistent in location but also exhibit distinctive internal structure by sex. These findings support the incorporation of fractal and complexity-based measures into national spatial epidemiology frameworks.

The entropy curves reveal several important nuances. Persistence maps display slightly higher entropy values across the full q-spectrum, particularly at the lowest and highest q-values. This indicates that persistent hotspots possess greater internal variability, encompassing both rare and dominant spatial structures. Among all groups, total population maps exhibit the widest entropy range, reflecting diverse and less predictable spatial organization. Sex-based differences, though subtle, are noteworthy: male maps show sharper slopes, indicating a faster entropy decay with increasing q and a tendency toward spatial dominance, while female maps present smoother transitions and higher mid-range entropy values, suggesting more balanced spatial heterogeneity.

Overall, this multiscale fractal analysis demonstrates that pediatric cancer hotspots in Romania are not only spatially persistent but also internally differentiated by sex. These findings support the inclusion of fractal and complexity-based measures in national spatial epidemiology frameworks.

In the next section, we move from the geometric and informational complexity of cancer prevalence maps to a statistical investigation of how persistence and continuity co-vary within and across population groups. Specifically, we assess the strength of association between these spatial indicators using Pearson correlation coefficients, thereby complementing the fractal insights with quantitative evidence and exploring links to industrial activity, pollution exposure, and healthcare accessibility.

### 3.6. Statistical Measure

To complement the spatial insights described above, we assessed how strongly persistence and continuity are linked within—and across—sex-specific cohorts. Table 5 summarizes the Pearson correlation coefficients.

Across the entire dataset, persistence and continuity were almost perfectly correlated (r = 0.95; *p* < 0.05), confirming that ATUs that return to the highest-prevalence quartile year after year are, in most cases, the same units that remain there for long uninterrupted stretches. The same near-perfect association was observed when the analysis was stratified by sex (female r = 0.95; male r = 0.96; all *p* < 0.05), reinforcing the idea that long-term hotspots are remarkably stable once established.

By contrast, correlations across sexs were markedly lower (ranging from 0.16 to 0.74, not significant or only weakly significant), suggesting that the intensity and stability of high prevalence can diverge between boys and girls within the same ATU. These weaker cross-sex links likely reflect sex-specific tumour profiles, biological susceptibilities, or differences in healthcare-seeking and reporting practices noted earlier.

Taken together with the persistence-continuity maps (Figure 4a–c and Figure 5a–c), these correlation results strengthen the case for targeted, long-term interventions in the small subset of ATUs that drive the national burden—while also highlighting the need for sex-disaggregated monitoring to capture subtler local dynamics.

### 3.7. The Dynamics of Case Numbers in Human Settlements with the Highest Rates of Pediatric Cancer (‰)

Zooming from the regional scale to individual settlements reveals a handful of extreme outliers whose prevalence trajectories depart sharply from national and regional trends. Table 6 lists the fifteen ATUs with the highest pediatric-cancer prevalence (‰) recorded at any point between 2008 and 2017.

Most began the decade with zero or near-zero cases, followed by sudden surges late in the study window:

- Boianu Mare (Bihor County) climbed from 0–3‰ in the early years to 130.43‰ in 2017, after an intermediate spike of 49‰ in 2013.
- Valea Salciei (Buzău) showed the single highest value observed—178.29‰ in 2015—after several years with no registered cases.
- Buești (Ialomița) registered no cases until 2012, then rose steadily to 68.75‰ by 2017.
- Similar late-period escalations were documented in Nădrag (Timiș), Ocna de Fier (Caraș-Severin) and Zorlențu Mare (Caraș-Severin), among others.

All of these localities display a steep, temporal “step-change” rather than the gradual trends seen in regional data—a pattern that may signal point-source environmental exposures (e.g., legacy mining, industrial emissions), improvements in local case detection, or demographic shifts such as in-migration of high-risk populations. Their geographic dispersion—ranging from the Western Carpathians to the Bărăgan lowlands—suggests multiple, context-specific drivers rather than a single nationwide factor.

Given their outlier status and the absence of continuous high prevalence in preceding years, these settlements warrant targeted interdisciplinary field investigations. Environmental sampling, health-service audits, and community-level socio-economic assessments could help determine whether the observed spikes reflect genuine prevalence increases, diagnostic artefacts, or transient exposures. Findings from such micro-scale studies would complement the broader persistence-continuity maps (Figure 4a–c and Figure 5a–c) and provide actionable evidence for local authorities and public-health planners.

## 4. Discussion

In this national, registry-based descriptive analysis of childhood cancer in Romania [2008–2017], we observed heterogeneous geographic patterns of incidence alongside expected age- and sex-specific distributions. The leading diagnostic groups followed international profiles (e.g., leukemias, CNS tumors, lymphomas), and temporal variation was present but modest at the national scale. These population-level patterns motivate targeted, hypothesis-driven follow-up work.

Our present study, which focuses specifically on pediatric cancer, provides significant advancements and novel evidence in three key areas:

*A.*  *Methodological Refinement and Enhanced Reproducibility:*

The most critical advancement is our simplified and more transparent methodological workflow. Instead of the complex ImageJ 1.54p-based image analysis, we calculated persistence and continuity directly from the epidemiological data using a formula-based approach in Excel before visualizing the results in GIS

Advantage: This method is more straightforward, user-friendly, and easily reproducible by other researchers, thereby increasing the scientific utility and accessibility of the spatial persistence/continuity framework.

*B.*  *Novel Application of Advanced Complexity Metrics:*

A major novel contribution of our study is the introduction of fractal and complexity analysis to characterize the spatial structure of cancer hotspots. We applied:

Higuchi 2D Fractal Dimension: To quantify the geometric complexity and irregularity of the spatial patterns.

Kolmogorov Complexity: To estimate the compressibility and inherent randomness of the spatial distribution.

Rényi Entropy: To assess heterogeneity across multiple scales, revealing how rare events (negative q-orders) or dominant clusters (positive q-orders) influence the overall pattern.

Novelty: This provides a deeper, quantitative understanding of the “texture” and internal structure of pediatric cancer clusters, going beyond simple location mapping to describe their spatial complexity, which can hint at different underlying etiological factors.

*C.*  *Focus on a Distinct, Understudied Population with Fine-Scale Analysis:*

By focusing exclusively on pediatric cancer (0–18 years), we investigated a population with etiologies fundamentally different from adult cancers, which are often linked to lifestyle factors. Our analysis at the fine scale of 3181 Administrative-Territorial Units (ATUs) allows us to detect hyper-localized hotspots in specific towns or villages—patterns that were entirely masked in the previous, regional-level analysis. This enables the generation of new hypotheses about localized environmental exposures or access-to-care issues specific to children.

Although male and female maps largely overlap geographically, differences in the number of persistent/continuous ATUs suggest a role for both shared regional factors and sex-specific biological or behavioral influences. These findings point to the need for further multidisciplinary research combining persistence-continuity analysis with pollution indices, socio-economic vulnerability metrics, and access-to-care assessments—essential steps toward targeted prevention, equitable pediatric oncology planning, and in-depth etiological inquiry.

Thus, regarding the relation to prior etiologic evidence: The geographic variability we observe may be consistent with known differences in parental, perinatal, and environmental determinants of childhood cancer. For leukemias—especially acute lymphoblastic leukemia in early childhood—prior studies implicate interactions between prenatal/early-life immune development and patterns of common infections (“delayed infection” hypotheses), as well as parental age and perinatal characteristics (e.g., birth weight). For embryonal tumors (e.g., neuroblastoma, Wilms tumor), prenatal influences are frequently discussed, including maternal health, medication, and potential environmental exposures. Ambient air pollution (e.g., PM and traffic-related pollutants), agricultural pesticides, and ionizing radiation have also been associated with selected pediatric cancers in international literature, alongside socioeconomic and diagnostic-access gradients that can shape observed incidence. While our data cannot test these mechanisms directly, the spatial heterogeneity we document is compatible with plausible differences in these contextual factors across regions.

Importantly, some of these hypotheses concern maternal and perinatal factors; however, our dataset is not linked to maternal records and cannot evaluate gestational exposures or pregnancy-related conditions.

The spatial and temporal patterns detailed in Section 3—including regional hotspots, sex-specific tumor profiles, and settlement-level spikes—provide the empirical foundation for the following discussion. Here, we interpret those findings in light of previous research, public health needs, and data-quality constraints.

Conventional cancer registries tend to aggregate data at national or regional levels, masking local disparities. By applying GIS-based persistence and continuity metrics, our study exposed entrenched hotspots in Central Transylvania and the Southeast/Bărăgan Plain, as well as episodic spikes in villages such as Boianu Mare and Valea Salciei. Similar fine-scale analyses in Poland, Serbia, and Bulgaria have likewise revealed industrial-belt or low-land clusters that are invisible in national statistics [29,30,31,32]. These converging results underscore the utility of spatial modelling for hypothesis generation, resource prioritization, and policy design.

Despite a modest national decline in prevalence (2008–2017), several regions displayed long-term persistence and continuity. Such stability suggests underlying structural drivers—industrial legacies, intensive agriculture, or limited pediatric-oncology capacity—that warrant multisectoral investigation. The pronounced sex gap in tumour typology (malignant tumors more common in boys; benign/uncertain tumors rising in girls) echoes international reports linking male susceptibility to hematological malignancies and female predilection for vascular lesions [33].

Romania’s estimated 69% survival rate for childhood cancer remains ~10 percentage points below Western Europe [34]. Spatially disaggregated registries, like the one piloted here, are essential to close that gap by enabling early detection and targeted outreach.

Mapping persistent hotspots allows health authorities to overlay environmental (air, soil, water pollution), socio-economic (income, education), and infrastructural (distance to oncology centers) layers, creating predictive risk models for proactive screening. Civil-society organizations—including Little People, Dăruiește Aripi, Renașterea, and Magicamp—already supply psychosocial support and are building a national pediatric-oncology registry with spatial dimensions [21,35,36,37]. Aligning our hotspot maps with these networks could accelerate case-finding, improve data completeness, and optimize resource allocation.

Our identification of stable clusters parallels findings from Italy’s Po Valley and Germany’s Ruhr region, where long-term pediatric-cancer persistence has been linked to industrial emissions and vehicular pollution [38,39]. Conversely, the episodic surges we observed in isolated Romanian settlements resemble patterns reported in rural Spain after agrochemical spills [40], suggesting that both chronic and acute exposures may be operative. Such analogues reinforce the need for combining environmental monitoring and molecular epidemiology to disentangle causal pathways.

This study identifies several Romanian settlements exhibiting extreme, localized spikes in pediatric cancer prevalence, diverging sharply from national trends. These outliers—including Boianu Mare, Valea Salciei, and Buești—show near-zero early rates followed by sudden surges (e.g., up to 178.29‰), suggesting temporal “step-changes.” Such patterns may indicate point-source environmental exposures, improved case detection, or demographic shifts. Geographically dispersed, these outliers likely have context-specific drivers. We recommend targeted field investigation environmental sampling, health-service audits, and socio-economic assessments—to determine causes and inform local public health action.

### 4.1. Limitations and Future Directions

This study was designed as a descriptive analysis; we therefore refrain from causal interpretation and focus on characterizing population-level patterns given the available data. First, the analysis was confined to the 2008–2017 period because pre-2008 reporting was fragmented; incorporating data from more recent years will help confirm current trends and support near-real-time surveillance. Second, the models did not yet include environmental or socio-economic covariates—such as pollution loads, household income, or proximity to specialized clinics—variables that could illuminate causal pathways once integrated with the hotspot maps. Third, aggregation at the ATU level, while useful for national coverage, may conceal sharp intra-urban disparities; moving to finer spatial units (e.g., census tracts or neighborhoods) would capture these micro-inequities. Fourth, individual-level risk factors—genetic predisposition, prenatal exposures, parental occupation—were unavailable, limiting direct causal inference. Finally, regional differences in diagnostic capacity and reporting quality, especially in rural areas with fewer pediatric-oncology services, raise the possibility of under- or over-estimation of prevalence. Addressing these gaps through expanded temporal coverage, multisource data linkage, higher-resolution mapping, and targeted field studies will strengthen the explanatory power of spatial epidemiology and sharpen its utility for Romanian pediatric-cancer control.

Regarding ecological design our analysis is ecological: cancer counts and rates were aggregated at the entrenched hotspots in (county/region) Central Transylvania and the Southeast/Bărăgan Plain, as well as episodic spikes in villages such as Boianu Mare and Valea Salciei level, and results pertain to populations rather than individuals. As such, ecological fallacy is possible; associations at the area level may not reflect individual-level relationships.

We also lacked harmonized regional covariates (e.g., migration, detailed socioeconomic structure, healthcare access metrics) for adjustment, which can influence observed spatial patterns in descriptive analyses.

As the present study looked at cancer cases diagnosed between 0–18 years old, maternal exposures and risk factors need to be discussed. We have no maternal linkage. The cancer registry data are not linked to maternal information, birth registries, or perinatal exposure records (e.g., maternal age, comorbidities, medications, smoking, occupational exposures), precluding direct examination of maternal or gestational risk factors.

An important limitation is reliance on prevalence measures instead of incidence. Prevalence integrates new case occurrence with survival duration, diagnostic/registration intensity, and population turnover, which can inflate or depress local rates independently of etiologic risk. As a result, observed spatial persistence and continuity might be partly driven by survivorship or data-system artifacts (e.g., lead-time or ascertainment bias). Because causal hypothesis generation requires clear temporal ordering between exposure and outcome, subsequent studies should analyze incident, newly diagnosed cancers recorded by the registry, geocoded at time of diagnosis. These analyses should employ per-son-time denominators and age/sex standardization and consider latency windows and sensitivity checks for population change. Such a design is better suited for formal hypothesis testing regarding exposure–disease relationships.

### 4.2. Future Research

Future research should therefore (i) link cancer data with environmental and demographic layers, (ii) conduct case–control studies in high-prevalence zones, (iii) update analyses with post-2017 records, and (iv) develop predictive GIS models to guide early detection and resource planning. Addressing these gaps will deepen insight into pediatric-cancer determinants and embed spatial epidemiology more firmly in Romanian health policy [41].

Spatial modelling thus transforms registry data into actionable intelligence, guiding equitable pediatric-oncology strategies and advancing the broader goal of cancer control in low- and middle-income European settings.

To evaluate etiologic hypotheses implicated by our descriptive patterns, analytic designs using individual-level data are needed—e.g., population-based case–control or cohort linkages that integrate cancer registry entries with birth records, maternal health and exposure information, environmental monitoring (air pollution, pesticides), and fine-scale socioeconomic indicators.

## 5. Conclusions

This nationwide GIS-based study adds a spatial lens to pediatric-cancer surveillance in Romania and provides three key contributions. First, it confirms that childhood cancer is driven by a multi-layered interplay of genetic susceptibility, environmental exposure, and health-system capacity. Romanian reports confirm regional inequalities in access to care—with pediatric oncology centers concentrated in Cluj-Napoca, Bucharest, and Iași—as well as disparities in detection and reporting, as highlighted by international evaluations such as EUROCARE-5. Although national prevalence declined modestly between 2008 and 2017, several regions—particularly the industrialized Central and Southeast zones—showed long-term persistence, and in some localities, dramatic end-period spikes. These patterns indicate that structural or environmental determinants remain unequally distributed and must be addressed through place-based interventions.

Second, the study introduces and operationalizes the paired indicators of persistence and continuity of persistence, revealing stable high-risk clusters that would have been obscured in aggregate statistics. Mapping these clusters pinpoints where screening, early-diagnosis programs, and oncology resources should be intensified. The analysis also uncovers sex-specific disparities: malignant tumors are still more common in boys, whereas benign or uncertain neoplasms have risen faster in girls—underlining the need for sexdisaggregated monitoring and researchThird, our results underscore the strategic value of integrating spatial modelling into national cancer-control plans. Persistent hotspots should be overlaid with environmental (e.g., pollution), demographic, and genomic layers to build multidimensional risk profiles and predictive tools. Close collaboration with governmental bodies, hospital networks, and non-governmental organizations—such as Little People, Dăruiește Aripi, Renașterea, and Magicamp—will be essential for maintaining up-to-date registries and translating spatial intelligence into equitable, child-centered health policy. Fractal and complexity measure could help in understanding the relation of spatial distribution of persistence and continuity of persistence for female, male and total population. In general, FAIR (Findable, Accessible, Interoperable and Reusable) principles should address the need to ensure that treatments are accessible to all patient populations, that trial designs are ethically sound, and that the benefits of cancer research are shared equitably across different groups [42]. 

In summary, while Romania has made progress in reducing the overall burden of pediatric cancer, geographically persistent pockets of elevated risk remain.

## Figures and Tables

**Table 1:** Classification of High-Risk Zones by Persistence and Continuity of Pediatric Cancer (2008–2017).

Class	Persistence (Years)	Continuity (Years)
0	0 (no persistence)	0 (no continuity)
1	1–3 (low)	1–3 (low)
2	4–6 (moderate)	4–6 (moderate)
3	7–9 (high)	7–9 (high)
4	10 (maximum)	10 (maximum)

**Table 2:** Ten-year progression of pediatric-cancer cases by ICD-10 code and sex (M = male, F = female, T = total).

COD	2008	2009	2010	2011	2012	2013	2014	2015	2016	2017	TOTAL
**MALIGNANT TUMORS**											
**C00-C96 T**	9329	8941	9112	9279	8662	8792	8777	7501	6889	7254	**84,536**
**C00-C96 F**	4012	3789	3803	4017	3740	3772	3759	3206	2864	3072	**36,034**
**C00-C96 M**	5317	5152	5309	5262	4922	5020	5018	4295	4025	4182	**48,502**
***Malignancies, declared or presumed to be primary, with specified locations except those of lymphoid, hematopoietic and related tissue***											
**C00-C75 T**	3897	3760	3896	4154	4093	4033	4226	3772	3580	3853	**39,264**
**C00-C75 F**	1685	1632	1681	1878	1827	1796	1869	1744	1584	1666	**17,362**
**C00-C75 M**	2212	2128	2215	2276	2266	2237	2357	2028	1996	2187	**21,902**
***Malignant tumors with poorly defined, secondary and unspecified areas***											
**C76-C80 T**	142	192	144	164	107	155	215	171	159	129	**1578**
**C76-C80 F**	66	82	72	36	35	55	100	59	83	74	**662**
**C76-C80 M**	76	110	72	128	72	100	115	112	76	55	**916**
***Malignant tumors of lymphoid, hematopoietic and related tissues***											
**C81-C95 T**	5290	4989	5072	4961	4462	4604	4336	3558	3150	3272	**43,694**
**C81-C95 F**	2261	2075	2050	2103	1878	1921	1790	1403	1197	1332	**18,010**
**C81-C95 M**	3029	2914	3022	2858	2584	2683	2546	2155	1953	1940	**25,684**
**OTHER TYPES OF TUMORS**											
**D00-D48 T**	6988	7092	7343	6907	6930	7072	7180	7411	7615	7315	**71,853**
**D00-D48 F**	3847	3974	4088	3888	3817	3874	3982	4178	4252	4116	**40,016**
**D00-D48 M**	3141	3118	3255	3019	3113	3198	3198	3233	3363	3199	**31,837**
**In situ *tumors***											
**D00-D09 T**	17	22	19	19	20	6	5	7	7	8	**130**
**D00-D09 F**	9	18	12	9	8	4	3	2	1	6	**72**
**D00-D09 M**	8	4	7	10	12	2	2	5	6	2	**58**
***Benign tumors***											
**D10-D36 T**	6179	6203	6274	5839	5784	5684	5750	5628	5799	5487	**58,627**
**D10-D36 F**	3439	3471	3538	3311	3195	3080	3169	3167	3224	3054	**32,648**
**D10-D36 M**	2740	2732	2736	2528	2589	2604	2581	2461	2575	2433	**25,979**
***Tumors with unpredictable evolution or unknown behavior***											
**D37-D48 T**	792	867	1050	1049	1126	1382	1425	1776	1809	1820	**13,096**
**D37-D48 F**	399	485	538	568	614	790	810	1009	1027	1056	**7296**
**D37-D48 M**	393	382	512	481	512	592	615	767	782	764	**5800**

**Table 3:** Temporal Persistence and Continuity of Pediatric Cancer Prevalence in Romanian ATUs (2008–2017).

Indicator	Low (1–3 Years)	Moderate (4–6 Years)	High (7–9 Years)	Maximum (10 Years)
Persistence	1391 ATUs	350 ATUs	82 ATUs	7 ATUs
Continuity	1583 ATUs	206 ATUs	34 ATUs	7 ATUs

**Table 4:** Complexity measures (Higuchi 2D, Kolmogorov complexity) computed on spatial maps of pediatric cancer persistence and continuity (2008–2017), by sex and total population.

Map	Higuchi_2D	Kolmogorov Complexity
Continuity_Female	2.356	1.858
Continuity_Male	2.363	1.724
Continuity_Total	2.356	2.272
Persistence_Female	2.356	1.858
Persistence_Male	2.363	1.724
Persistence_Total	2.358	2.272

**Table 5:** Pearson correlations between persistence and continuity indicators. (Note. Pearson correlation coefficients; * *p* < 0.05).

	Total Persistence	Total Continuity	Female Persistence	Female Continuity	Male Persistence	Male Continuity
Total persistence	1.00	0.95 *	0.68 *	0.65 *	0.74 *	0.70 *
Total continuity	0.95 *	1.00	0.64 *	0.64 *	0.71 *	0.72 *
Female persistence	0.68 *	0.64 *	1.00	0.95 *	0.19	0.16
Female continuity	0.65 *	0.64 *	0.95 *	1.00	0.17	0.15
Male persistence	0.74 *	0.71 *	0.19	0.17	1.00	0.96 *
Male continuity	0.70 *	0.72 *	0.16	0.15	0.96 *	1.00

**Table 6:** Dynamics of pediatric-cancer prevalence (‰) in highest-risk settlements, 2008–2017.

	County	Human Settlement	2008	2009	2010	2011	2012	2013	2014	2015	2016	2017
1	BIHOR	BOIANU MARE	0.00	2.79	0.00	32.74	21.15	49.08	37.85	16.08	9.93	130.43
2	ALBA	SOHODOL	0.00	2.73	2.74	0.00	0.00	0.00	0.00	0.00	35.71	93.28
3	BUZĂU	VALEA SALCIEI	6.17	0.00	0.00	0.00	0.00	0.00	0.00	178.29	0.00	76.92
4	IALOMIȚA	BUEȘTI	0.00	0.00	0.00	0.00	5.38	0.00	0.00	0.00	0.00	68.75
5	TIMIȘ	NĂDRAG	0.00	1.78	1.81	0.00	0.00	0.00	5.91	7.98	20.83	51.33
6	CLUJ	RECEA-CRISTUR	0.00	0.00	3.46	6.71	0.00	3.13	0.00	0.00	2.82	46.32
7	DOLJ	APELE VII	0.00	0.00	0.00	4.43	4.56	2.36	47.15	27.10	16.48	45.58
8	TIMIȘ	BALINT	0.00	0.00	0.00	0.00	3.08	34.27	16.29	28.75	35.60	43.48
9	CARAȘ-SEVERIN	OCNA DE FIER	0.00	0.00	0.00	0.00	0.00	0.00	6.85	0.00	0.00	42.55
10	BUZĂU	CERNĂTEȘTI	1.40	7.10	0.00	0.00	0.00	3.07	14.59	9.92	6.69	40.35
11	IALOMIȚA	GRIVIȚA	1.28	0.00	20.16	18.89	4.16	7.18	30.75	36.64	23.26	42.14
12	HARGHITA	SĂCEL	0.00	0.00	0.00	3.08	0.00	3.10	0.00	0.00	3.16	39.09
13	SUCEAVA	CÎRLIBABA	0.00	2.16	0.00	0.00	0.00	0.00	2.35	0.00	0.00	38.28
14	CARAȘ-SEVERIN	ZORLENȚU MARE	0.00	0.00	0.00	0.00	0.00	0.00	0.00	75.63	138.46	17.09
15	SIBIU	CHIRPAR	0.00	0.00	0.00	4.14	2.14	2.16	0.00	15.80	44.60	26.89

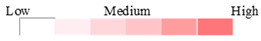
 Source: *Ministry of Health*.

## Data Availability

The data presented in this study are available on request from the corresponding author. The data are not publicly available due to privacy.
